# Fracture Load of 3D-Printed Interim Three-Unit Fixed Dental Prostheses: Impact of Printing Orientation and Post-Curing Time

**DOI:** 10.3390/polym15071737

**Published:** 2023-03-31

**Authors:** Reem I. Alkhateeb, Hadeel S. Algaoud, Rand B. Aldamanhori, Rand R. Alshubaili, Haidar Alalawi, Mohammed M. Gad

**Affiliations:** 1 College of Dentistry, Imam Abdulrahman Bin Faisal University, P.O. Box 1982, Dammam 31441, Saudi Arabia; 2160003471@iau.edu.sa (R.I.A.); 2160003060@iau.edu.sa (H.S.A.); 2160004111@iau.edu.sa (R.B.A.); 2160003145@iau.edu.sa (R.R.A.); 2 Department of Substitutive Dental Sciences, College of Dentistry, Imam Abdulrahman Bin Faisal University, P.O. Box 1982, Dammam 31441, Saudi Arabia; haalalawi@iau.edu.sa

**Keywords:** 3D printing, interim fixed dental prostheses, fracture resistance, mechanical testing, post-curing time, printing orientation

## Abstract

The fracture resistance of 3-unit interim fixed dental prostheses (IFDPs) fabricated using digital light processing (DLP) additive technology with different printing parameters is neglected. Therefore, this study investigates the effect of different printing orientations and different post-curing times on the fracture resistance of 3-unit IFDPs fabricated from two three-dimensional (3D) printed resins, NextDent, C&B (CB), ASIGA, and DentaTOOTH. A 3-unit dye was scanned, and an IFDP was designed. A total of 300 specimens (150/materials, *n* = 10) were printed and divided into three groups according to printing orientations (0°, 45°, 90°) per material. Each orientation was subdivided into five groups (*n* = 10) considering the post-curing time (green state as control, 30, 60, 90, and 120 min). All specimens underwent thermocycling (5000 cycles). Each specimen was fitted onto the die and loaded until fracture using a universal testing machine with a loading rate of 1 m/min. Data were analyzed using ANOVA and post hoc Tukey test (α = 0.05). The result showed that printing orientation had a significant effect on the fracture load for both ASIGA and NextDent materials (*p* < 0.05). The highest fracture load was recorded with 45° orientation, followed by 0° orientation and 90° orientation showed the lowest values per respective post-curing time. Post-curing time increased the fracture load (*p <* 0.05). Post-curing time had a positive effect on the fracture load. As the post-curing time increased, the fracture resistance load increased (*p* < 0.05), with 90 and 120 min showing the highest fracture load. The 0° and 45° printing orientations have a high fracture load for 3D-printed IFDPs, and an increased post-curing time is recommended.

## 1. Introduction

The interim fixed dental prostheses (IFDPs) are critical components of fixed prosthodontics as they aid in the ground plan for the design of a fixed dental prosthesis [1]. IFDPs have many functions, such as protecting the pulpal and periodontal tissue, and helping in the healing process by guiding the tissue healing to achieve a satisfactory emergence profile [2]. Furthermore, it helps assess oral hygiene procedures, preserves space by preventing the migration of abutments, and provides an acceptable occlusal scheme [3].

IFDPs can be divided into two types: prefabricated or custom-made. Prefabricated examples include stock aluminum cylinders, anatomic metal crowns, clear celluloid shells, and polycarbonate crown forms, which are usually used for a single tooth. However, in fixed partial dentures, custom-made bridges are used, and they can be fabricated by direct or indirect techniques using different types of acrylic resins [1]. The provisional material selected is based on mechanical, physical, and handling properties. Other important factors include biocompatibility and complications from intraoral use [4]. Interim materials were classified into two types: composite resin-based (aromatic/aliphatic dimethacrylate) and polymer-based (poly methyl methacrylate, PMMA) [5]. Polymethyl methacrylate PMMA resin, polyethyl methacrylate (PEMA) resin, polyvinyl methacrylate resin, bis-acryl composite resin, and visible light-cured urethane dimethacrylates are among the materials commonly used for custom IFDPs [6,7].

Computer-aided design/computer-aided manufacturing (CAD/CAM) has numerous applications in clinical dentistry. It can be used in multiple specialties, including prosthodontics, oral maxillofacial surgery, implantology, and orthodontics [8,9]. CAD/CAM is used in the prosthodontics field via two fabrication methods: the subtractive (SM) and additive method (AM) [10,11,12]. In the SM method, the IFDPs are milled from prefabricated blocks using different milling machines and technologies [8,9,13]. Meanwhile, in AM, the IFDPs are built layer by layer using 3D printable photo-polymerized fluid resins [1,11,14]. AM has some advantages over SM, such as using fewer materials with almost no material loss. Moreover, it can print multiple materials with favorable detailed reproduction [8]. According to the literature, the advantages of using 3D printing technology to fabricate IFDPs include being more economical than the conventional method, reducing the carbon footprint, and helping save energy and materials [15]. Another study stated that 3D printing provides high-quality restorations with easy and fast fabrication [16].

The performance of IFDPs varies in the literature, owing primarily to printing parameters and polymer compositions [17]. Henderson found that 3D-printed bis-acrylic-based resin has low flexural strength when compared to conventional bis-acrylic-based resins [18]. Low flexural strength was observed with 3D-printed microhybrid resin [6] and UDMA-based resins [19] when compared to PMMA-based CAD-CAM milled interim prostheses. Previous studies [20,21,22] compared the fracture strength of interim prostheses to CAD-CAM milled and conventional resins using PMMA-based 3D-printed resins. Suralik et al. [22] reported high fracture strength of 3D printed vs. conventional resin and bis-acrylic resins, as reported by Reymus et al. [20]. Mayer et al. [19] reported low fracture strength of 3D-printed resin vs. CAD/CAM milled resins [23]. According to Reeponmaha et al. [24], MMA-based 3D-printed resins have higher fracture strength than PMMA-based CAD/CAM milled and conventional resins.

Taşn et al. [9] and Crenn et al. [25] demonstrated that 3D-printed resin had higher strength than conventional MMA and bis-acrylic-based resins in the case of composite-based 3D-printed interim reins. Whereas 3D printed strength was reported to be lower when compared to PMMA-based CAD-CAM milled [26] and conventional heat cured [27]. Simoneti et al. [19] reported that conventional resins have high strength. Although the strength of 3D-printed resins varies with composition, improving strength through printing parameter modifications was suggested [28], and one ester-based and one PMMA-based polymer were chosen for this study. However, due to limited information on ASIGA composition, comparing both materials based on material compositions was difficult.

Despite the fact that polymer-based resins have been widely used and investigated for 3D-printed interim prostheses, the composition of 3D-printed resins is limited due to either novelty or company limitations (not completely disclosed by manufacturers such as ASIGA resin). Pantea et al. [7] proposed that 3D-printed resins appear to be classified in the same way as conventional IFDPs materials. Al-Qahtani et al. [29] compared 3D-printed resins with different compositions and discovered that the strength of printed reins was dependent on chemical composition, with 3D-printed urethane methacrylate and printed acrylic ester resin improving strength when compared to light-cured resin. Different monomer types in 3D-printed resin compositions affect the physical and mechanical properties of the printed object [23,30]. Digholkar et al. [26] demonstrated high flexural strength and microhardness in 3D-printed bis-acryl composite resins with cross-linked monomers and inorganic fillers.

When using 3D printing, numerous factors in terms of printing parameters should be considered that might influence the printed objects’ properties [28]. Among these factors are printing orientations [31], post-curing time [32], and printing technology [8,33,34]. Printing orientation is said to influence the mechanical properties of 3D-printed specimens in several previous studies. In a previous study [35], comparing the fracture resistance of IFDPs fabricated using light curing resin with different printing orientations, including occlusal, vertical, palatal, and diagonal, it was concluded that the diagonal printing orientation was superior in fracture resistance [35]. Another study [31] tested two 3D-printed resins, NextDent and Detax, with different printing orientations (0°, 45°, 90°) and found that the Detax at 90° orientation showed the highest flexure strength while 45° showed the minimum values. Additionally, these finding was material-type dependent, as NextDent showed close values at 0° and 90° [31].

Another important printing parameter to consider when using 3D-printing technology is post-curing time, which can impact the mechanical properties of the printed specimen [32]. A previous study compared the flexural properties of 3D-printed bar-shaped specimens with different post-curing times and concluded that strength increased when post-cured for 60–90 min [32]. Another study [36] investigated the effects of post-curing time on the mechanical properties, hardness, biocompatibility, and degree of conversion of 3D-printed resin and concluded that increasing the post-curing time improves the flexural properties, hardness, and biocompatibility [36]. Reymus et al. [20] compared different 3D-printed resins for IFDPs with building orientation (long-axis positioned either occlusal, buccal, or distal to the printer’s platform) at different post-curing times. They found an association between tested parameters and the strength of the printed object and concluded that increased post-curing time was associated with an increase in fracture resistance [20].

Although the effect of printing orientation and post-curing time on the fracture resistance of 3D-printed IFDPs have been investigated in a few studies, almost all studies investigated both factors separately on bar-shaped specimens. Therefore, this study aims to compare the effect of printing orientations and post-curing time, as well as the combined effect of both factors, on the fracture load of 3D-printed three-unit IFDPs, which has not been investigated yet. The null hypothesis states that there is no significance in the fracture load of 3D-printed IFDPs printed with different orientations and different post-curing times.

## 2. Materials and Methods

This study’s sample size was calculated based on previous study findings [14], with a 5% error margin and 80% study power. Ellakany et al. reported the facture load ± SD of milled, 3D printed ASIGS, 3D-printed NextDent, and conventional methods as 1794.06 ± 34.83, 1067.57 ± 91.85, 1720.26 ± 71.18, and 1008.23 ± 62.87, respectively. Using sample size calculation [37] and comparing the means, it was determined that 10 specimens per group are required for a total of 300 specimens (150/resin, *n* = 10/group) using two different 3D-printed resins (NextDent and ASIGA).

### 2.1. Specimen Fabrication

**Figure 1:**
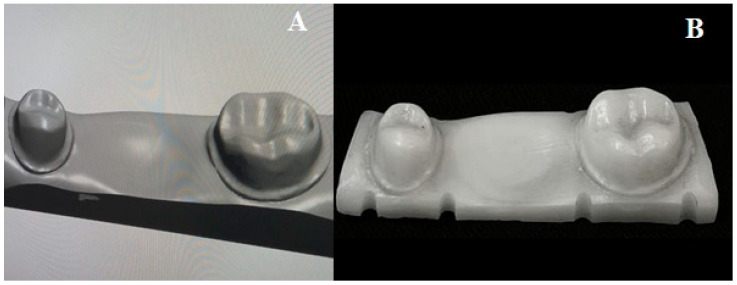
STL file for die (**A**) and the milled zirconia die for specimen testing (**B**) with scale bar 5 mm.

**Figure 2:**
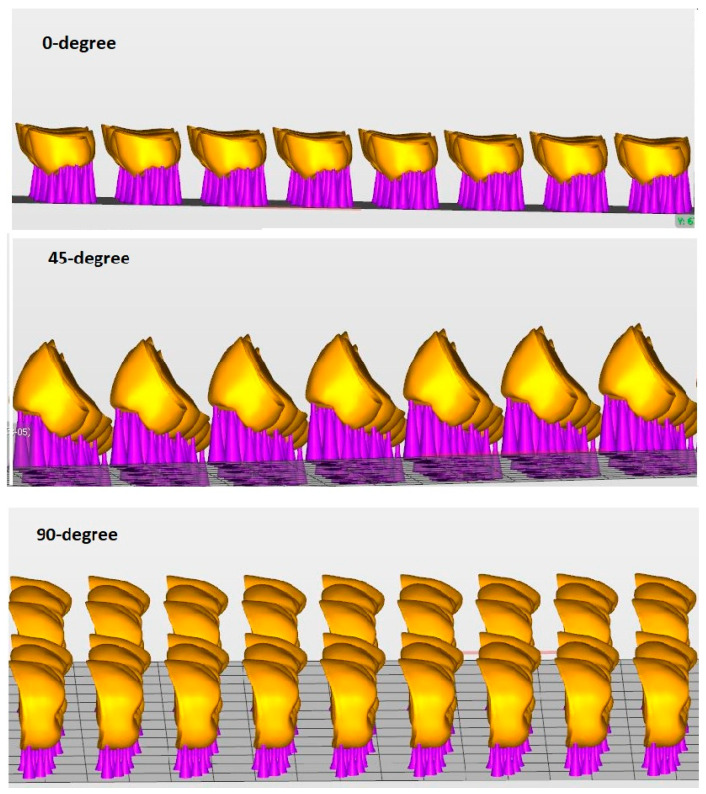
Specimen design with different printing orientations with scale bar 5 mm.

**Figure 3:**
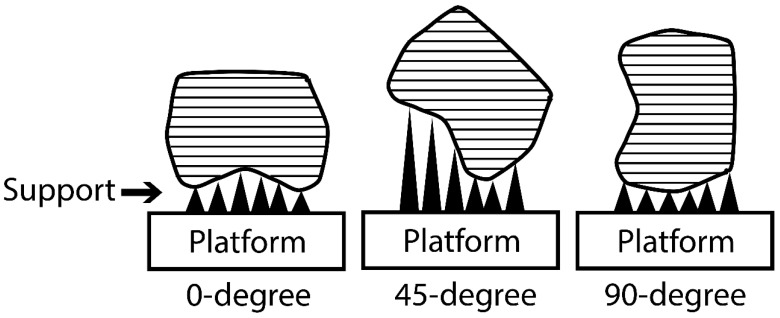
An illustrated diagram of the printed layers with different printing orientations for the 3D-printed IFDP specimens.

Table 1 summarizes the materials used in this study and their specifications for fabrication. A three-unit zirconia die (Figure 1) was prepared to receive the mandibular 3-unit bridge that was fabricated with premolar and molar abutments with the recommended amount of reduction for final zirconia restoration. Both abutments were prepared with a chamfer finish line, with an axial reduction of 1 mm and an occlusal reduction of 1.5 mm. The die was scanned using an intraoral scanner (TRIOS 3, 3shape, Copenhagen, Denmark), and a standard tessellation language file (STL) was created. Using the recommended settings of the 3Shape Dental System (3shape, Copenhagen, Denmark), a three-unit IFDP was designed with connector sizes of 15.05 mm² mesial to the first molar and 14.07 mm² distal [35] (Figure 2 and Figure 3). The three-unit IFDPs were then printed using the NextDent (3D Sprint software, 3D Systems, Rock Hill, SC, USA) and ASIGA (Asiga Composer, Asiga, Alexandria, Australia) resin printing systems, as described in Table 1.

**Figure 4:**
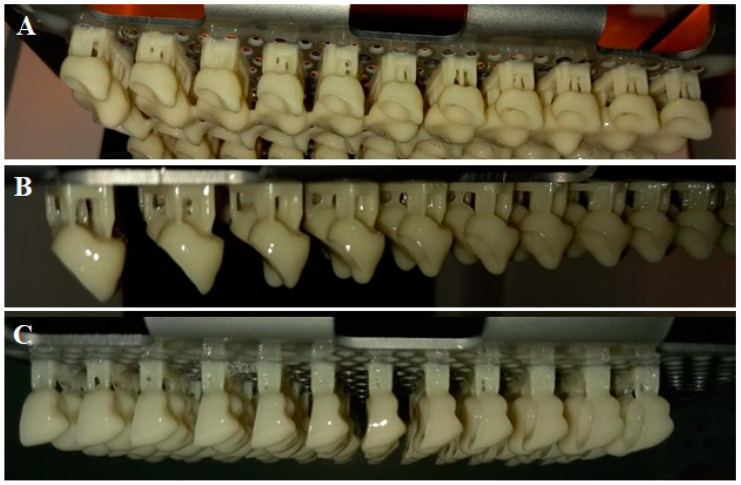
(**A**–**C**). The 3D-printed specimens with different orientations for NextDent (**A**) 0-degree, (**B**) 45-degree, and (**C**) 90-degree (scale bar 5 mm).

**Figure 5:**
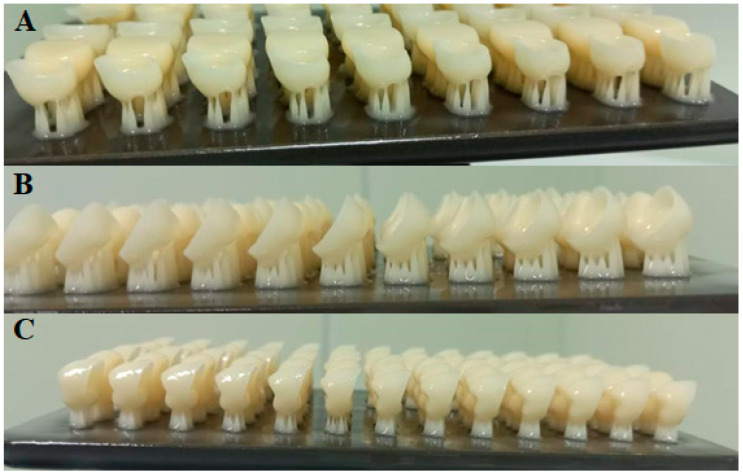
The 3D-printed specimens with different orientations for ASIGA. (**A**) 0-degree, (**B**) 45-degree, and (**C**) 90-degree (scale bar 5 mm).

According to the printing orientation, each material was printed with different printing orientations 0°, 45°, and 90° (Figure 4 and Figure 5). After printing the specimens, 99% Isopropyl alcohol was used to clean printed specimens from un-polymerized fluid resin. According to the post-curing time, each orientation group was subdivided into five groups (*n* = 10) based on post-curing time; green state (GS, no post-curing), 30, 60, 90, 120 min, as described in Table 1. After support removal, all specimens were finished and inspected for any defects, cracks, or chipping. The approved specimens were stored in distilled water at 37 °C for 24 h [14,20] and then subjected to thermal cycling for 5000 cycles (5 °C to 55 °C) with a dowel time of 30 s using a thermocycling machine (THE-1100 Thermocycler, SD Mechatronik GMBH, Pleidelsheim, Germany).

**Figure 6:**
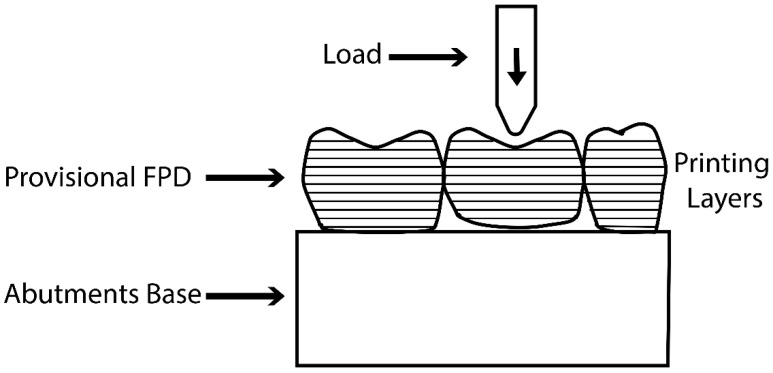
Prepared specimen with the provisional prosthesis loaded on the Instron machine (0-degree group).

For the fracture load test, each specimen was fitted on the die, which was stabilized on the customized plate of a universal testing machine (Instron Model 8871; Instron Corp., Norwood, MA, USA). A metal indenter was loaded onto the 3-unit IFDPs exactly at the central fossa of the pontic to create a tripod contact and standardize the test for all specimens (Figure 6). The indenter was attached to a 5 kN load cell, and each specimen was then loaded to failure at a loading rate of 1 m/min [20]. The fracture pattern and position were assessed by two evaluators who determined the site of fracture (at connector, pontic, or crown) as well as the direction of fracture (vertical or horizontal, segmental/layered, or multiple fractures) [14,35,38]. The specimens fractured in five different modes, which were classified according to the shape and position of the fracture site: (I) crown or chipped or fractured into small pieces, (II) connector fracture, (III) pontic fracture, and (IV) layering fracture.

### 2.2. Statistical Analysis

The JMP^®^ statistical analysis software (Version <16>. SAS Institute Inc., Cary, NC, USA, 1989–2021) was used for data analysis. Means and standard deviations were calculated for the descriptive data analysis. The normality of the data was tested using the Shapiro–Wilk test, and the results showed that the data were non-normally distributed. Three-way ANOVA and paired Student *t*-tests were used to test for significant variation in average fracture resistance due to different materials, print angles, and post-curing times. All *p*-values less than 0.05 were considered statistically significant.

## 3. Results

Descriptive statistics, including mean, standard deviation, minimum, and maximum values, were generated for the variable in tested groups (Table 2 and Table 3). The mean fracture load levels with post-curing times ranged between 1041.01 ± 145.87 N and 1487.58 ± 179.52 N for ASIGA groups and 980.72 ± 298.70 N and 1683.56 ± 207.57 N for the NextDent groups. In general, NextDent groups had a significantly and statistically higher fracture load compared to ASIGA groups (*p* < 0.0001). For ASIGA resin with different post-curing times, the 45° print angle significantly had a higher fracture load, followed by the 0° print angle, while the 90° angle showed the lowest fracture load. No significant difference in fracture load between 0° and 45° print angles with all post-curing times except the 60-min groups (*p* = 0.004). The highest fracture load was recorded with 45° (1487.58 ± 179.52 N) and 90° (1434.43 ± 288.52 N) print angles at 120 min. For NextDent resin with different post-curing times, both 0° and 45° print angles showed significantly high fracture load when compared with the 90° print angle (*p* < 0.005) except 0° vs. 90° at 60 min (*p* = 0.092). No significant difference in fracture load between 0° and 45° print angles with all post-curing times (*p* > 0.05). The highest fracture load was found with 0° (1683.56 ± 207.57 N) and 45° (1507.19 ± 90.37 N) print angles at 120 min.

Regardless of the print angle, 0-min (no post-curing) groups showed the lowest fracture load per respective printing angle (*p* < 0.0001). Increased post-curing time in reads the fracture load, and this increase was directly propositional to increased fracture load when the post-curing time was more than 60 min (*p* < 0.0001). The highest fracture load values were recorded at the 120 min mark for three print angles: 45° followed by 0°, and 90° the lowest for ASIGA, and 0° followed by 45°; 90° was the lowest for NextDent.

The majority of specimens with a 90° print angle showed the lowest values. However, ASIGA 0° print angle was lowest in value with both 30 and 60 min of post-curing time (1013.31 ± 140.13 N and 1067.35 ± 75.42 N), respectively. Whereas NextDent only varied in 0° print angle presented with the lowest value among 30 min of post-curing time (980.72 ± 298.70 N). The groups with a 0° print angle and 120 min post-curing time in both tested materials displayed the highest fracture load. The NextDent material, 0 and 45° print angle and 120 min post-curing time showed statistically significant higher fracture resistance compared to ASIGA material in 0° (*p* = 0.0009) and 45° (*p* = 0.0034). On the other hand, the lowest fracture load was observed with the groups with a 90° print angle and 0 min of post-curing time in both tested materials. Interestingly, the NextDent material, at 0° print angle and 0 min of post-curing time, showed statistically significantly lower fracture resistance compared to ASIGA material at 0° print angle and 0 min post-curing time (*p* = 0.0068).

**Figure 7:**
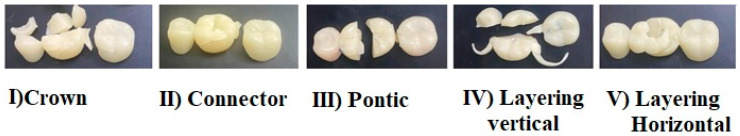
Representative photos displaying the type and position of different fractures.

Table 4 summarizes the pattern of specimen failure according to the position and extent of specimen fracture. The green state groups showed type I, where almost all specimens were completely fractured into small pieces with crowns fractured. For Type II and type III (Connector and pontics), there were variations between groups, with the 0° and 45° angles showing the most dominance. Meanwhile, the 90° angle showed the most dominance in terms of layering fracture for both materials, except for the ASIGA 0° 30 min and 120 min groups, which showed a horizontal layering fracture (Figure 7). Horizontal layering fracture (IV) was mostly seen in ASIGA 0° orientation with 60, 90, and 120 min, representing half of the specimens, or 50% of the groups. Longitudinal layering fracture (V) was seen in both NextDent and ASIGA, but was mostly related to 90° orientation with 30, 60, 90, and 120 post-curing time, representing 52.5% and 47.5% of the specimens, respectively.

## 4. Discussion

This study compared the fracture resistance of 3D printed 3-unit IFDPs fabricated from two 3D printed resins, NextDent and ASIGA, in terms of printing orientations and the effect of post-curing time. The study’s null hypothesis was that there would be no difference in fracture load of NextDent and ASIGA printed IFDPs with different printing orientations and post-curing times, as both printing parameters affected the fracture load.

Thermocycling was performed on the materials tested to expose them to fatigue and simulate the oral conditions [28]. This process created stresses corresponding to the mechanical stresses in the mouth of IFPDs due to the abrupt change in temperature when they are submerged in the baths [39]. According to ISO 11405, thermal aging for 10,000 cycles simulates 1 year of clinical use [40,41]. In the present study, all IFDPs were subjected to 5000 cycles of thermal cycling, approximately simulating six months of clinical usage of IFDPs. The 3D-printed resins absorb water when immersed in water, and water uptake increases as the water temperature increases [41]. Due to the polarity of resin materials, water can penetrate through the bulk of the resin and force monomers and other additives to diffuse out [42]. Additionally, water fills the interpolymeric chain spaces, forces the polymer chains apart, reduces the intermolecular force to the weaker form of bonds known as “van der Waals bonds”, initiates cracks within the material, induces bulk swelling, and eventually lowers the resin’s mechanical performance [42,43]. With aging, some components leach out, and the entrance of water results in swelling, hydrolysis, and degradation of the cross-linked polymer. All these have negative effects on the mechanical properties [40,41,44]. Therefore, thermal cycling was used as an aging procedure for all specimens before fracture testing.

In oral environments, IFDPs are subjected to different stresses, mainly vertical occlusal forces [2]. The direction of the applied load may affect the strength of printed objects due to the printing nature (layer-by-layer) of additively manufactured IFDPs [45]. In this study, a 3-unit bridge configuration was selected instead of the bar-shape specimen, mimicking the clinical conditions of PFPs [14,20]. With different printing orientations, the layering direction changed according to the load direction [20]. For example, at 0°, the applied load is perpendicular to the printing layer direction [30], and this is expected to increase the strength of the printed object [20,40]. Clinically, with 0°, the support is located at the occlusal surface, and this might affect the occlusal surface with support removal [14,40]. Therefore, different printing orientations were suggested to improve the strength and functional stability of the occlusal surface.

Reymus et al. [20] compared different 3D-printed resins for IFDPs with building orientations at the occlusal (0°) buccal or distal to the printing platform, but these two orientations (buccal and distal) made the layer direction parallel to the applied load and might affect the strength [20]. Therefore, in the present study, different orientations in relation to the long axis of the printed bridge were selected, keeping the layer direction approximately perpendicular to the load direction (Figure 3). The only exception was 90°, which was longitudinal instead of cross-sectional.

Regardless of the post-curing time, both 0° and 45° orientations showed high fracture loads of IFDPs. This can be attributed to the direction of load in relation to the printing layer directions, which is obvious in the 0° orientation. In the 45° orientation, the printing layers are in an oblique direction instead of parallel, which may also explain the increased fracture load [38,40]. This finding is in agreement with previous studies [38,46,47,48]. Turksayar et al. [38] reported the highest fracture strength in the 0° orientation (1094.80 N), which is close to the 30 min groups with all printing orientations.

In Reymus’s study [20], different orientations were used, with the long axis positioned either occlusal, buccal, or distal to the printer’s platform. Occlusal and buccal orientations are the same as the 0 and 90° orientations, respectively, in the present study. The third orientation was distal, with the specimens printed vertically from the distal surface of the molar to the mesial surface of the molar. This orientation made the printing layer direction parallel to the load direction [47]. Additionally, the removal of supporting structures at the distal surface of the molar may affect the contact between the first and second molars. Therefore, the 90° orientation was replaced by the 45° orientation.

In the present study, the load direction was made parallel to the printing layer (longitudinal direction along 3-unit bridge length) at a 90 degree. Although no significant difference was observed, the lowest values were recorded at 90 degrees per post-curing time. With load application, splitting of some of the specimens along the printing layer direction (Figure 7) was observed, which was linked with poor interlayer bonding [20]. It was found that interlayer bonding was weaker than bonding within the layer itself [46], and the same concept was reported with KEßLER [40] regarding the vertical direction, which makes the load parallel to the printed layer direction. It was reported that the adhesion between each two sequentially printed layers could be affected by the printed object’s different orientations [20]. Alharbi et al. [46] demonstrated that the incremental layer-by-layer printing technology initiated crack propagation and was considered the main concern in structural failures of 3D-printed resins. However, this finding is in disagreement with Nold et al. [35] and Unkovskiy et al. [49], who reported that the vertical orientations showed the highest flexural strength. The variation may be attributed to the tested specimen configurations (bar-shape instead of the 3-unit bridge) and differences in aging procedures.

After printing, the specimens are in a green state and contain uncured monomer, which requires further polymerization in accordance with the manufacturer’s recommendations regarding the post-curing process [20,28,32,48,49]. Each company has its specific time required to ensure even polymerization of printed objects, which depends on the printing technology and curing device [20,28,50]. In a previous study by Kim et al. [32], different post-curing times were used to evaluate the degree of conversion, and it found that as the post-curing time increased, the degree of conversion also increased compared to the green state group [32].

One way to improve the resin’s mechanical properties is to cross-link the polymer chains to prevent sliding against each other [51]. In the present study, the 3D-printed resin is UV-light activated, resulting in the polymerization and cross-linking of the bulk of components; bisphenol A-glycidil dimethacrylate or urethane dimethacrylate [52]. However, some residual monomer remains uncured and requires further post-curing processing to achieve full-structure cross-linking, thus completing the polymerization and improving the mechanical properties [11,36,53]. This is consistent with the results of this study; when comparing all groups to the green state group, the lowest fracture load was observed in the green state group. Additionally, the green state group displayed a type I fracture, where it chipped and fractured into small pieces.

Regardless of printing orientation, the fracture load increased significantly as post-curing times increased for both materials. This could be explained based on the increased degree of monomer conversion when subjected to more post-curing time [28,54,55]. It was reported that the amount of residual monomer affects the strength of printed materials, and once the post-polymerization treatment started, the residual monomer decreased due to monomer conversion [32]. Previous studies [32,36] have investigated the degree of conversion of printed resins with different post-curing times and found that all post-cured groups showed a high degree of conversion in comparison to the green state group. Additionally, a higher degree of conversion was attained with a high degree of polymerization and resulted in an improvement in the strength of printed resin [49,50,56,57,58]. This is in agreement with the finding of the present study in terms of the post-curing time effect; post-curing times of 90 and 120 min showed higher fracture load, and this increase is proportional to time; as time increases, the strength increases. The results of this study are consistent with different studies [32,36,48,49] that investigated the effect of post-curing time on the strength of 3D-printed resins and reported a positive impact of increased post-curing time. Kim et al. [32] investigated the strength of 3D-printed IFDPs with different post-curing times up to 90 min and reported that flexural properties increased when specimens were post-cured for a longer time. Bayarsaikhan et al. [36] also reported an increase in the strength of 3D-printed IFDPs bar-shaped specimens with an increased post-curing time of up to 120 min [48,59]. In the present study, the post-curing time was extended up to 120 min and had a positive impact on the fracture load of IFDPs. 

Previous studies [6,38,45] investigated fracture analysis using different methods. Nold et al. [11] classified the fracture position into crown, pontic, and both connectors, while Turksayar et al. [38] analyzed it based on crack or fracture incidence and whether it was catastrophic or non-catastrophic. In the present study, the layering fracture type was observed and added to our classification. The layering type might reflect the effect of printing layer direction according to printing orientations. The authors expected that the layering fracture would be correlated with the printing orientations, regardless of post-curing time. However, the distribution of layering between groups was approximately equal. The variation was related to the direction of failure (horizontal or vertical/splitting along specimen length), which could be correlated with printing orientation. The vertical one was dominant at a 90° angle, confirming weak interlayer bonding. Turksayar et al. [38] reported catastrophic failure in connectors, pontic, and crowns but did not report the layering. This may be due to the variation in methods, post-curing time, and the mold used for specimen testing (metal die with cylindrical abutment).

The masticatory force in the posterior region is around 350 N [60] and may increase up to 900 N [57]. Therefore, IFDPs with a high fracture load are required to withstand the masticatory forces without fracture. All fracture loads of the tested groups are above 900 N except for the green state. So, for long-term temporization, IFDPs with high strength are required, especially for patients who have higher masticatory force than normal, such as in patients with bruxism [38].

From the clinical point of view, the 0° and 45° support positions have more clinical applicability in terms of support position and removal, in addition to the high fracture resistance compared to the 90° angle. On the other hand, the post-curing time has a primary role and is considered the main factor affecting the strength, followed by printing orientation [28]. Therefore, post-curing methods must be considered as a primary factor among factors affecting the strength of printed IFDPs. With any printing orientation, an increased post-curing time of up to 120 min is recommended, regardless of the material type. Even if we use a 0° printing angle, it is better to increase the post-curing time. Finally, when selecting the material type, the printing parameters must be considered, especially post-curing conditions and time.

Although this study had several strengths, including the use of different resins and printing parameters, as well as thermal aging, the lack of cyclic loading is considered a limitation of the present study. Furthermore, the study was conducted in an artificial environment that could not fully replicate the oral environment. In addition, the fracture load in a clinical setting may differ from that in the in vitro model. Therefore, future studies are needed to investigate further the effect of printing orientation and post-curing time on the 3D-printed IFDPs under conditions that simulate oral conditions, with different time intervals and cyclic loading. Additionally, future studies recommended could include the use of one or a combination of other printing parameters, such as the layer thickness and post-curing temperature [28]. Future in vivo studies could also be conducted to substantiate the findings of the present study.

## 5. Conclusions

This study was performed to assess and compare the effect of printing orientations and post-curing time on the fracture resistance of 3D-printed 3-unit interim fixed dental prostheses. The printing orientations affect the strength of 3D-printed 3-unit interim fixed dental prostheses. The fracture resistance of 3D-printed 3-unit interim fixed dental prostheses increased with 0° and 45° printing orientations. In terms of post-curing time factors, the fracture load significantly increased as post-curing time increased, and the highest fracture load was achieved at 120 min. With any printing orientation, the post-curing time has a greater effect on fracture resistance; thus, priority should be given to post-curing time. Interim fixed dental prostheses with high strength could be printed by changing the printing orientation to 0 or 45 degrees and increasing the posturing time to 120 min. However, more research is needed with longer post-curing times of more than 120 min and degree of conversion considerations with long-span (4-unit) interim fixed dental prostheses.

## Figures and Tables

**Table 1:** Specifications and fabrication methods of materials used in the present study.

Material/Brand Name	Composition	Printer/Printing Technology	Layer Thickness	Printing Orientations	Post-Curing Machine	Post-Curing Time	Post-Curing Temperature
NextDent C&B (CB) NextDent, Soesterberg, The Netherlands	Microfilled hybrid methacrylic acid ester-based resin >60% wt methacrylic oligomer (UDMA, EGDMA), 15–25% wt HEMA	Next Dent 5100 Digital Light Processing (DLP)	50 µm	0°, 45°, 90°	Machine: LC-D Print Box Wavelength: 405 nm	GS, 30, 60, 90, and 120	60 °C
ASIGA Asiga DentaTOOTH (ASIGA, Erfurt, Germany)	Methacrylate-based Microhybrid composite resin	ASIGA MAX™ LED-based Digital Light Processing (DLP)	50 µm	0°, 45°, 90°	Machine: Asiga Flash Wavelength: 405 nm	GS, 30, 60, 90, and 120 min	60 °C

GS, green state.

**Table 2:** Fracture load (N) across the different tested groups for ASIGA resin.

Orientation	Post-Curing Time					*p*
	0 min	30 min	60 min	90 min	120 min	
0	794.83 ± 68.52	1013.31 ± 140.13 ^a,A^	1067.35 ± 75.42 ^a,A^	1267.00 ± 240.58 ^A^	1434.43 ± 288.52 ^A^	<0.001
45	626.32 ± 96.41 ^A^	1113.47 ± 61.84 ^a,A^	1102.81 ± 148.05 ^a^	1327.30 ± 161.96 ^A^	1487.58 ± 179.52 ^A^	<0.001
90	602.03 ± 82.76 ^A^	1041.01 ± 145.87 ^a^	1076.02 ± 89.74 ^a,A^	1124.87 ± 121.59	1203.05 ± 114.49	<0.001
*p*	<0.001	0.003	0.005	0.002	<0.001	

Same small letter per raw indicating non-significant between groups (*p* < 0.05). Same capital letter per column indicating insignificant between groups (*p* < 0.05).

**Table 3:** Fracture load (N) across the different tested groups for NextDent resin.

Orientation	Post-Curing Time					*p*
	0 min	30 min	60 min	90 min	120 min	
0	610.06 ± 208.95	980.72 ± 298.70	1259.64 ± 205.80 ^A^	1476.99 ± 71.47 ^A^	1683.56 ± 207.57 ^A^	<0.001
45	532.83 ± 109.21 ^A^	1307.32 ± 88.89	1438.88 ± 209.60 ^a^	1437.02 ± 230.00 ^a,A^	1507.19 ± 90.37 ^A^	<0.001
90	503.29 ± 196.37 ^A^	1168.46 ± 172.91	1207.51 ± 151.98 ^a,A^	1237.17 ± 98.03 ^a^	1342.44 ± 76.05	<0.001
*p*	0.032	<0.001	0.013	0.0012	<0.001	

Same small letter per raw indicating non-significant between groups (*p* < 0.05). Same capital letter per column indicating insignificant between groups (*p* < 0.05).

**Table 4:** Mode of specimens fractures according to shape and position of fracture site.

Materials	Orientation	Post-Curing Time																								
		GS					30 min					60 min					90 min					120 min				
		I	II	III	IV	N	I	II	III	IV	N	I	II	III	IV	N	I	II	III	IV	N	I	II	III	IV	N
NextDent	0	6	2	2	-	10	3	3	3	1	10	2	4	-	4	10	-	5	4	1	10	-	4	3	3	10
	45	4	1	1	4	10	1	1	2	6	10	2	2	1	5	10	2	1	2	5	10	-	3	1	6	10
	90	5	1	-	4	10	2	2	-	6	10	1	2	2	5	10	-	2	2	6	10	-	3	1	6	10
ASIGA	0	7	2	1	-	10	1	3	1	5	10	-	2	3	5	10	-	3	2	5	10	-	2	2	6	10
	45	8	1	1	-	10	3	1	2	4	10	1	3	2	4	10	1	3	2	4	10	-	2	4	4	10
	90	6	1	-	3	10	2	1	-	7	10	-	-	4	6	10	0	3	2	5	10	1	1	3	5	10

I: Crown (Chipped and fractured into small pieces). II: Connector. III: Pontic. IV: Layering.

## Data Availability

The data are available upon request via email or phone to the corresponding author.

## References

[1] Kadiyala K.K., Badisa M.K., Anne G., Anche S.C., Chiramana S., Muvva S.B., Zakkula S., Jyothula R.R. (2016). Evaluation of Flexural Strength of Thermocycled Interim Resin Materials Used in Prosthetic Rehabilitation—An In-vitro Study. J. Clin. Diagn. Res..

[2] Burns D.R., Beck D.A., Nelson S.K. (2003). A review of selected dental literature on contemporary provisional fixed prosthodontic treatment: Report of the Committee on Research in Fixed Prosthodontics of the Academy of Fixed Prosthodontics. J. Prosthet. Dent..

[3] Patras M., Naka O., Doukoudakis S., Pissiotis A. (2011). Management of Provisional Restorations’ Deficiencies: A Literature Review. J. Esthet. Restor. Dent..

[4] Hahnel S., Krifka S., Behr M., Kolbeck C., Lang R., Rosentritt M. (2019). Performance of resin materials for temporary fixed denture prostheses. J. Oral Sci..

[5] Greuling A., Matthies A., Eisenburger M. (2023). Fracture load of 4-unit interim fixed partial dentures using 3D-printed and traditionally manufactured materials. J. Prosthet. Dent..

[6] Abad-Coronel C., Carrera E., Córdova N.M., Fajardo J.I., Aliaga P. (2021). Comparative Analysis of Fracture Resistance between CAD/CAM Materials for Interim Fixed Prosthesis. Materials.

[7] Pantea M., Ciocoiu R.C., Greabu M., Ripszky Totan A., Imre M., Țâncu A.M.C., Sfeatcu R., Spînu T.C., Ilinca R., Petre A.E. (2022). Compressive and Flexural Strength of 3D-Printed and Conventional Resins Designated for Interim Fixed Dental Pros-theses: An In Vitro Comparison. Materials.

[8] Tian Y., Chen C., Xu X., Wang J., Hou X., Li K., Lu X., Shi H., Lee E.-S., Jiang H.B. (2021). A Review of 3D Printing in Dentistry: Technologies, Affecting Factors, and Applications. Scanning.

[9] Ta¸sın S., Ismatullaev A. (2022). Comparative evaluation of the effect of thermocycling on the mechanical properties of conventionally polymerized, CAD-CAM milled, and 3D-printed interim materials. J. Prosthet. Dent..

[10] Sadid-Zadeh R., Zirkel C., Makwoka S., Li R. (2021). Fracture Strength of Interim CAD/CAM and Conventional Partial Fixed Dental Prostheses. J. Prosthodont..

[11] Revilla-León M., Meyers M.J., Zandinejad A., Özcan M. (2019). A review on chemical composition, mechanical properties, and manu-facturing work flow of additively manufactured current polymers for interim dental restorations. J. Esthet. Restor. Dent..

[12] Kessler A., Hickel R., Reymus M. (2020). 3D Printing in Dentistry—State of the Art. Oper. Dent..

[13] Huettig F., Prutscher A.M.I., Goldammer C., Kreutzer C.A., Weber H. (2015). First clinical experiences with CAD/CAM-fabricated PMMA-based fixed dental prostheses as long-term temporaries. Clin. Oral Investig..

[14] Ellakany P., Fouda S.M., Mahrous A.A., AlGhamdi M.A., Aly N.M. (2022). Influence of CAD/CAM Milling and 3D-Printing Fabrication Methods on the Mechanical Properties of 3-Unit Interim Fixed Dental Prosthesis after Thermo-Mechanical Aging Process. Polymers.

[15] Park S.-M., Park J.-M., Kim S.-K., Heo S.-J., Koak J.-Y. (2020). Flexural Strength of 3D-Printing Resin Materials for Provisional Fixed Dental Prostheses. Materials.

[16] Zaharia C., Gabor A.G., Gavrilovici A., Stan A.T., Idorasi L., Sinescu C., Negruțiu M.L. (2017). Digital dentistry-3D printing applications. J. Interdiscip. Med..

[17] Jain S., Sayed M.E., Shetty M., Alqahtani S.M., Al Wadei M.H.D., Gupta S.G., Othman A.A.A., Alshehri A.H., Alqarni H., Mobarki A.H. (2022). Physical and Mechanical Properties of 3D-Printed Provisional Crowns and Fixed Dental Prosthesis Resins Compared to CAD/CAM Milled and Conventional Provi-sional Resins: A Systematic Review and Meta-Analysis. Polymers.

[18] Henderson J.Y., Korioth T.V., Tantbirojn D., Versluis A. (2022). Failure load of milled, 3D-printed, and conventional chair-side-dispensed interim 3-unit fixed dental prostheses. J. Prosthet. Dent..

[19] Mayer J., Stawarczyk B., Vogt K., Hickel R., Edelhoff D., Reymus M. (2021). Influence of cleaning methods after 3D printing on two-body wear and fracture load of resin-based temporary crown and bridge material. Clin. Oral Investig..

[20] Reymus M., Fabritius R., Keßler A., Hickel R., Edelhoff D., Stawarczyk B. (2019). Fracture load of 3D-printed fixed dental prostheses compared with milled and conventionally fabricated ones: The impact of resin material, build direction, post-curing, and artificial aging—An in vitro study. Clin. Oral Investig..

[21] Ibrahim A., Shehawy D.E., El-Naggar G. (2020). Fracture resistance of interim restoration constructed by 3D printing versus CAD/CAM technique (in vitro study). Ain Shams Dent. J..

[22] Suralik K., Sun J., Chen C.-Y., Lee S. (2020). Effect of Fabrication Method on Fracture Strength of Provisional Implant-Supported Fixed Dental Prostheses. Prosthesis.

[23] Wuersching S.N., Hickel R., Edelhoff D., Kollmuss M. (2022). Initial biocompatibility of novel resins for 3D printed fixed dental prostheses. Dent. Mater..

[24] Reeponmaha T., Angwaravong O., Angwarawong T. (2020). Comparison of fracture strength after thermo-mechanical aging be-tween provisional crowns made with CAD/CAM and conventional method. J. Adv. Prosthodont..

[25] Crenn M.J., Rohman G., Fromentin O., Benoit A. (2022). Polylactic acid as a biocompatible polymer for three-dimensional print-ing of interim prosthesis: Mechanical characterization. Dent. Mater. J..

[26] Madhav V.N.V., Digholkar S., Palaskar J. (2016). Evaluation of the flexural strength and microhardness of provisional crown and bridge materials fabricated by different methods. J. Indian Prosthodont. Soc..

[27] Tahayeri A., Morgan M., Fugolin A.P., Bompolaki D., Athirasala A., Pfeifer C.S., Ferracane J.L., Bertassoni L.E. (2018). 3D printed versus conventionally cured provisional crown and bridge dental materials. Dent. Mater..

[28] Gad M.M., Fouda S.M. (2023). Factors affecting flexural strength of 3D-printed resins: A systematic review. J. Prosthodont..

[29] Al-Qahtani A.S., Tulbah H.I., Binhasan M., Abbasi M.S., Ahmed N., Shabib S., Farooq I., Aldahian N., Nisar S.S., Tanveer S.A. (2021). Surface Properties of Polymer Resins Fabricated with Subtractive and Additive Manufacturing Techniques. Polymers.

[30] Choi Y., Yoon J., Kim J., Lee C., Oh J., Cho N. (2022). Development of Bisphenol-A-Glycidyl-Methacrylate- and Trimethylolpro-pane-Triacrylate-Based Stereolithography 3D Printing Materials. Polymers.

[31] Derban P., Negrea R., Rominu M., Marsavina L. (2021). Influence of the Printing Angle and Load Direction on Flexure Strength in 3D Printed Materials for Provisional Dental Restorations. Materials.

[32] Kim D., Shim J.-S., Lee D., Shin S.-H., Nam N.-E., Park K.-H., Shim J.-S., Kim J.-E. (2020). Effects of Post-Curing Time on the Mechanical and Color Properties of Three-Dimensional Printed Crown and Bridge Materials. Polymers.

[33] Moradi M., Aminzadeh A., Rahmatabadi D., Hakimi A. (2021). Experimental investigation on mechanical characterization of 3D printed PLA produced by fused deposition modeling (FDM). Mater. Res. Express.

[34] Moradi M., Aminzadeh A., Rahmatabadi D., Rasouli S.A. (2021). Statistical and Experimental Analysis of Process Parameters of 3D Nylon Printed Parts by Fused Deposition Modeling: Response Surface Modeling and Optimization. J. Mater. Eng. Perform..

[35] Nold J., Wesemann C., Rieg L., Binder L., Witkowski S., Spies B.C., Kohal R.J. (2021). Does Printing Orientation Matter? In-Vitro Fracture Strength of Temporary Fixed Dental Prostheses after a 1-Year Simulation in the Artificial Mouth. Materials.

[36] Bayarsaikhan E., Lim J.-H., Shin S.-H., Park K.-H., Park Y.-B., Lee J.-H., Kim J.-E. (2021). Effects of Postcuring Temperature on the Mechanical Properties and Biocompatibility of Three-Dimensional Printed Dental Resin Material. Polymers.

[37] Kadam P., Bhalerao S. (2010). Sample size calculation. Int. J. Ayurveda Res..

[38] Turksayar A.A.D., Donmez M.B., Olcay E.O., Demirel M., Demir E. (2022). Effect of printing orientation on the fracture strength of additively manufactured 3-unit interim fixed dental prostheses after aging. J. Dent..

[39] Almeida C.S., Amaral M., Gonçalves F.D.C.P., Paes-Junior T.J.D.A. (2015). Effect of an experimental silica-nylon reinforcement on the fracture load and flexural strength of bisacrylic interim partial fixed dental prostheses. J. Prosthet. Dent..

[40] Keßler A., Hickel R., Ilie N. (2021). In vitro investigation of the influence of printing direction on the flexural strength, flexural modulus and fractographic analysis of 3D-printed temporary materials. Dent. Mater. J..

[41] Gale M., Darvell B. (1999). Thermal cycling procedures for laboratory testing of dental restorations. J. Dent..

[42] Hamanaka I., Iwamoto M., Lassila L., Vallittu P., Shimizu H., Takahashi Y. (2014). Influence of water sorption on mechanical properties of injection-molded thermoplastic denture base resins. Acta Odontol. Scand..

[43] Alzaid M., AlToraibily F., Al-Qarni F.D., Al-Thobity A.M., Akhtar S., Ali S., Al-Harbi F.A., Gad M.M. (2022). The Effect of Salivary pH on the Flexural Strength and Surface Properties of CAD/CAM Denture Base Materials. Eur. J. Dent..

[44] Par M., Tarle Z., Hickel R., Ilie N. (2018). Mechanical properties of experimental composites containing bioactive glass after artificial aging in water and ethanol. Clin. Oral Investig..

[45] Alshamrani A.A., Raju R., Ellakwa A. (2022). Effect of Printing Layer Thickness and Postprinting Conditions on the Flexural Strength and Hardness of a 3D-Printed Resin. BioMed Res. Int..

[46] Alharbi N., Osman R., Wismeijer D. (2016). Effects of build direction on the mechanical properties of 3D-printed complete coverage interim dental restorations. J. Prosthet. Dent..

[47] Park S.M., Park J.M., Kim S.K., Heo S.J., Koak J.Y. (2019). Comparison of flexural strength of three-dimensional printed three-unit provisional fixed dental prostheses according to build directions. J. Kor. Dent. Sci..

[48] Al-Dulaijan Y.A., Alsulaimi L., Alotaibi R., Alboainain A., Akhtar S., Khan S.Q., Al-Ghamdi M., Gad M.M. (2022). Effect of printing orientation and post-curing time on the flexural strength of 3D-printed resins. J. Prosthodont..

[49] Unkovskiy A., Bui P.H.-B., Schille C., Geis-Gerstorfer J., Huettig F., Spintzyk S. (2018). Objects build orientation, positioning, and curing influence dimensional accuracy and flexural properties of stereolithographically printed resin. Dent. Mater..

[50] Reymus M., Lümkemann N., Stawarczyk B. (2019). 3D-printed material for temporary restorations: Impact of print layer thickness and post-curing method on degree of conversion. Int. J. Comput. Dent..

[51] Saen-Isara T., Dechkunakorn S., Anuwongnukroh N., Srikhirin T., Tanodekaew S., Wichai W. (2017). Influence of the cross-linking agent on mechanical properties of PMMA powder with compromised particle morphology. Int. Orthod..

[52] Vitale A., Cabral J.T. (2016). Frontal conversion and uniformity in 3D printing by photopolymerisation. Materials.

[53] Perea-Lowery L., Gibreel M., Vallittu P.K., Lassila L.V. (2021). 3D-Printed vs. Heat-Polymerizing and Autopolymerizing Denture Base Acrylic Resins. Materials.

[54] Song G., Son J.-W., Jang J.-H., Choi S.-H., Jang W.-H., Lee B.-N., Park C. (2021). Comparing volumetric and biological aspects of 3D-printed interim restorations under various post-curing modes. J. Adv. Prosthodont..

[55] Calheiros F.C., Daronch M., Rueggeberg F.A., Braga R.R. (2008). Degree of conversion and mechanical properties of a BisGMA:TEGDMA composite as a function of the applied radiant exposure. J. Biomed. Mater. Res. Part B Appl. Biomater..

[56] dos Santos R.L., de Sampaio G.A., de Carvalho F.G., Pithon M.M., Guênes G.M., Alves P.M. (2014). Influence of degree of conversion on the biocompatibility of different composites in vivo. J. Adhes. Dent..

[57] Perea-Lowery L., Gibreel M., Vallittu P.K., Lassila L. (2020). Evaluation of the mechanical properties and degree of conversion of 3D printed splint material. J. Mech. Behav. Biomed. Mater..

[58] Jin G., Gu H., Jang M., Bayarsaikhan E., Lim J.H., Shim J.S., Lee K.W., Kim J.E. (2022). Influence of post washing process on the elution of residual monomers, degree of conversion, and mechanical properties of a 3D printed crown and bridge materials. Dent. Mater..

[59] Li P., Lambart A.-L., Stawarczyk B., Reymus M., Spintzyk S. (2021). Postpolymerization of a 3D-printed denture base polymer: Impact of post-curing methods on surface characteristics, flexural strength, and cytotoxicity. J. Dent..

[60] Pihut M., Wisniewska G., Majewski P., Gronkiewicz K., Majewski S. (2009). Measurement of occlusal forces in the therapy of functional disorders with the use of botulinum toxin type A. J. Physiol. Pharmacol. Off. J. Pol. Physiol. Soc..

